# Removing Short Wavelengths From Polychromatic White Light Attenuates Circadian Phase Resetting in Rats

**DOI:** 10.3389/fnins.2019.00954

**Published:** 2019-09-10

**Authors:** Bojana Gladanac, James Jonkman, Colin M. Shapiro, Theodore J. Brown, Martin R. Ralph, Robert F. Casper, Shadab A. Rahman

**Affiliations:** ^1^Lunenfeld-Tanenbaum Research Institute, Mount Sinai Hospital, Toronto, ON, Canada; ^2^Institute of Medical Sciences, University of Toronto, Toronto, ON, Canada; ^3^Advanced Optical Microscopy Facility, University Health Network, Toronto, ON, Canada; ^4^Department of Psychiatry and Ophthalmology, University of Toronto, Toronto, ON, Canada; ^5^Youthdale Child and Adolescent Sleep Centre, Toronto, ON, Canada; ^6^Division of Reproductive Endocrinology and Infertility, University of Toronto, Toronto, ON, Canada; ^7^Department of Psychology, University of Toronto, Toronto, ON, Canada; ^8^Division of Sleep and Circadian Disorders, Departments of Medicine and Neurology, Brigham and Women’s Hospital, Boston, MA, United States; ^9^Division of Sleep Medicine, Harvard Medical School, Boston, MA, United States

**Keywords:** circadian phase resetting, pacemaker, light, locomotor activity, spectral tuning

## Abstract

Visible light is the principal stimulus for resetting the mammalian central circadian pacemaker. Circadian phase resetting is most sensitive to short-wavelength (blue) visible light. We examined the effects of removing short-wavelengths < 500 nm from polychromatic white light using optical filters on circadian phase resetting in rats. Under high irradiance conditions, both long- (7 h) and short- (1 h) duration short-wavelength filtered (< 500 nm) light exposure attenuated phase-delay shifts in locomotor activity rhythms by (∼40–50%) as compared to unfiltered light exposure. However, there was no attenuation in phase resetting under low irradiance conditions. Additionally, the reduction in phase-delay shifts corresponded to regionally specific attenuation in molecular markers of pacemaker activation in response to light exposure, including c-FOS, Per1 and Per2. These results demonstrate that removing short-wavelengths from polychromatic white light can attenuate circadian phase resetting in an irradiance dependent manner. These results have important implications for designing and optimizing lighting interventions to enhance circadian adaptation.

## Introduction

Circadian misalignment is associated with long-term health problems ranging from depression (Hickie and Rogers, 2011; Rajaratnam et al., 2011) to diabetes (Morris et al., 2015). Repeated changes in the light-dark schedule, as is common in shift work, can induce circadian misalignment (James et al., 2007; Skene et al., 2018). Light is the strongest environmental time cue for synchronizing the endogenous pacemaker in the suprachiasmatic nuclei (SCN) (Duffy et al., 1996; Czeisler and Gooley, 2007) and controlling the light-dark schedule can attenuate circadian misalignment (Czeisler et al., 1990; Santhi et al., 2005; Santhi et al., 2007). One of the major operational challenges in controlling the light-dark schedule is avoiding light exposure at inappropriate times. For example, night shift workers returning home after their night shift are exposed to light during the day, which can attenuate entrainment to a night time schedule (Boivin and James, 2002; Smith et al., 2008; Sasseville and Hebert, 2010; Boivin et al., 2012). However, modulating the characteristics of light such as intensity and spectrum may provide a mechanism to control the disruptive effects of aberrant light exposure on circadian rhythms.

Non-image forming (NIF) responses to light, which includes circadian phase resetting and acute changes in hormone levels, are mediated by an integrated neuronal pathway originating at the retina. Intrinsically photosensitive retinal ganglion cells (ipRGCs) are the principal photoreceptors mediating this response under bright long-duration light exposure conditions (Guler et al., 2008; Gooley et al., 2010), as is common during shift work. These melanopsin-expressing ipRGCs are preferentially short-wavelength sensitive (λ_*max*_ ∼480 nm), and NIF responses to light mediated by these photoreceptors, including melatonin suppression and phase resetting, also show corresponding short-wavelength sensitivity (peak sensitivity range ∼ 450–480 nm) (Takahashi et al., 1984; Boulos, 1995; Brainard et al., 2001; Thapan et al., 2001; Lockley et al., 2003; Warman et al., 2003; Cajochen et al., 2005; Lockley et al., 2006; Gooley et al., 2010; Bedrosian et al., 2013). Moreover, previous studies have shown that melanopsin knockout mice have preserved but significantly reduced photic-induced phase resetting (Panda et al., 2002; Ruby et al., 2002). Therefore, NIF responses to light may be modulated by controlling the short-wavelength content of broad-spectrum white light.

Removing wavelengths shorter than ∼500 nm (0% transmission) from broad-spectrum white light can attenuate the suppression of melatonin during nocturnal light exposure (Kayumov et al., 2005; Sasseville et al., 2006; Rahman et al., 2008; Sasseville and Hebert, 2010; Rahman et al., 2011; van der Lely et al., 2015; Gil-Lozano et al., 2016; Rahman et al., 2017; Regente et al., 2017; Souman et al., 2018), and prevent alterations in central and peripheral clock gene expression (Rahman et al., 2008, 2011). However, the effect of filtering these photic wavelengths on circadian phase resetting has not yet been determined. While melatonin suppression and circadian phase resetting are often coincidental, they are functionally decoupled such that phase resetting can occur even without melatonin suppression (Zeitzer et al., 1997, 2011; Paul et al., 2009; Kiessling et al., 2014; Rahman et al., 2018). Although one prior study suggests that filtering short-wavelengths < 520 nm may attenuate circadian phase shifts in humans exposed to light at night during a simulated night shift (Regente et al., 2017), methodological limitations preclude clear conclusions. Therefore, we examined whether circadian phase resetting induced by light exposure is affected by modulating the spectral composition of broad-spectrum white light. We hypothesized that removing short-wavelengths < 500 nm (blue portion of the visible spectrum) from polychromatic light would attenuate phase-delay shifts induced by nocturnal light exposure. Additionally, we examined the effects of filtering short-wavelengths < 500 nm on SCN activation to identify the temporal and spatial neural pathway mediating the changes in phase resetting magnitude. Since the relative contribution of the photoreceptors depends on intensity and duration of exposure, we explored whether the phase resetting responses to short-wavelength filtered light differed between short (1 h) and long (7 h) duration exposures and bright (100 μW/cm^2^) and dim (10 μW/cm^2^) exposures.

## Materials and Methods

### Animals

Male Sprague Dawley rats weighing between 200–250 g were obtained from Charles River Laboratories (Charles River Laboratories, Saint Constant, QC, Canada). Animals were individually housed in cages equipped with stainless steel running wheels (MiniMitter, Bend, OR, United States), and food and water was available *ad libitum*. Cages were placed in open stainless-steel chambers. Animals were entrained to 12:12 LD for at least 2 weeks before being released into constant darkness (DD). Uniform illumination distribution [98.3 ± 7.2 μW/cm^2^ (∼500 lux) at cage level] was provided by Philips cool white fluorescent lights (4100K) placed facing the housing chambers. All procedures were approved by the University of Toronto Animal Care Committee and were in accordance with the guidelines established by the Canadian Council on Animal Care.

### Light Source

**FIGURE 1:**
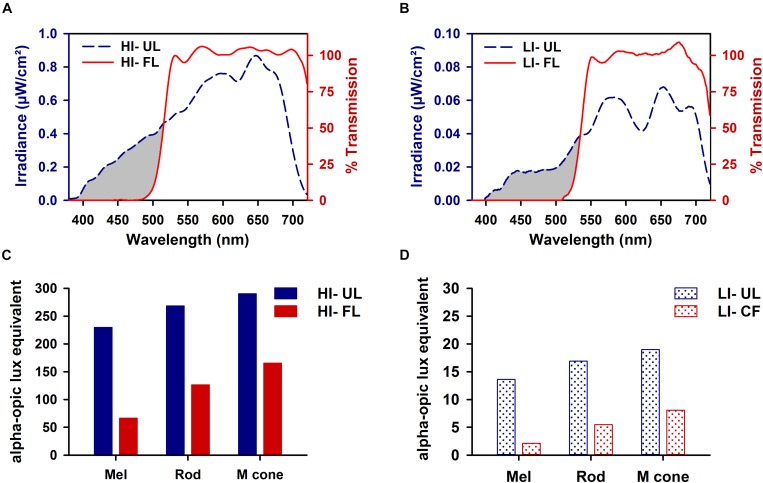
Spectral transmission profiles of high irradiance (HI ∼100 μW/cm^2^; **A**) and low irradiance (LI ∼10 μW/cm^2^; **B**) filtered polychromatic light. Transmission profile of filtered light (FL; red line) shown against the tungsten-halogen unfiltered light source (UL; blue dashed line) display the removal of all visual short wavelengths < 500 nm (represented by the gray-shaded region). Corresponding representation of melanopsin (λ_*max*_ 480 nm), rod opsin (λ_*max*_ 498 nm), and M-cone opsin (λ_*max*_ 508 nm) photoreceptor activity produced under HI **(C)** and LI **(D)** FL and UL conditions.

The top shelf of the stainless-steel housing chamber was constructed to have a controlled lighting environment and was used exclusively for light exposure episodes. Illumination was provided by light from a fiber optic FS-7L illuminator (Magic Lite Ltd., Mississauga, ON, Canada) fitted with a 183-W tungsten halogen MR-16 bulb (3000K, General Electric) that was transmitted by six FSPT-50 fiber optic bundles (Magic Lite Ltd.). Each FSPT-50 bundle consisted of 50 random sorted end-emitting fiber optic cables. The illuminator was located outside the animal-housing chamber in a custom ventilated box that prevented light from escaping the illuminator except through the fiber optic bundles. One or two animal cages were exposed to experimental lighting provided by three fiber optic bundles positioned above each cage. Spectral output was measured using a National Institute of Standards calibrated Ocean Optics S2000 spectroradiometer (Dunedin, FL, United States). Light was filtered at the source using a cut-off filter that removed all transmission < 500 nm (FL; Figures 1A,B; red lines) (Zircadium Inc., Toronto, ON, Canada). Neutral density filters (Kodak, Rochester, NY, United States) were used to lower irradiance from high irradiance (HI, ∼100 μW/cm^2^; Figure 1A) to low irradiance (LI, ∼10 μW/cm^2^; Figure 1B). The Rodent Toolbox v1.0 was used to quantify contributions of different photoreceptors of the rodent retina under different spectral conditions (Lucas et al., 2014).

### Behavioral Experiments

#### Circadian Phase Shift Protocol

Wheel-running activity was continuously recorded using VitalView software (Philips-Respironics, Bend, OR, United States). After entrainment, animals were maintained in constant darkness (DD) for at least 2 weeks. All animals were handled in DD with the aid of night vision equipment (American Technologies Network Corp., San Francisco, CA, United States). Cage changes in DD were performed with the aid of a red-light lamp (Kodak LED Safelight; Kodak, Rochester, NY, United States). Free-running animals were exposed to filtered light (FL) or unfiltered light (UL) for 1 h at the high or low irradiance level starting at circadian time (CT) 16. Free-running animals were also exposed to FL or UL for 7 h at the high irradiance level starting at CT13. Dark control animals were handled in the same manner but remained in darkness.

#### Estimation of Circadian Phase Shifts

Following photic stimuli, animals were maintained for an additional 2 weeks in DD. Activity records were analyzed using Clocklab software package (Actimetrics, Evanston, IL, United States) in 6 min bins. Activity onset under DD was used as the circadian phase marker. Data were averaged across at least 10 days before and after the stimulus by linear regression through activity onsets. To minimize possible confounding due to transient phase shifts, activity onset data from up to 3 days immediately after the stimulus were not included in the regression analysis. To objectively minimize possible confounding of activity onsets due to cage changes and food/water maintenance, activity onsets that were not within 30 min of the regression line were excluded. Out of a total of 1,426 activity onsets estimated, 149 were excluded. The magnitude of phase shift was calculated as the extrapolated difference between the two linear regressions the day after the light pulse. By convention, negative values represent phase delays and positive values represent phase advances.

### Immunohistochemistry (IHC) and *in situ* Hybridization (ISH)

#### Tissue Preparation

Animals were sacrificed at 0, 2, and 8 h after the end of the 1-h exposure condition. Animal handling and tissue sample collection were performed under darkness using a safe red-light lamp (Kodak LED Safelight), and with the aid of night vision equipment (American Technologies Network Corp.). Animals were anesthetized with Isoflurane and perfused transcardially with 120 ml of 0.9% saline followed by 300–350 ml of 4% (w/v) paraformaldehyde (PFA)/PBS solution. Brains were then post-fixed in the same fixative, submerged in 30% (w/v) sucrose/PBS cryoprotectant solution and stored frozen at −80°C in Tissue-Tek OCT compound (Sakura Finetek, Torrance, CA, United States). For future orientation, brain segments were nicked in the dorsal region of the left hemisphere and then sliced into 40-μm thick coronal sections using a cryostat (Leica CM3050S, Leica Microsystems, Wetzler, Germany). Identification of the SCN was based on key landmarks including the development and shape of the optic chiasm, and the decussation of the anterior commissure. Sections were stored in cryoprotectant at −20°C until further processing.

#### Immunohistochemistry

Serial free-floating sections (1:4) were rinsed in PBS and incubated in 5% (v/v) normal goat serum, 0.3% (v/v) Triton X-100 in PBS for 1 h prior to overnight primary antibody incubation at 4°C. All serial sets were stained for double-labeled fluorescence in 0.2% (v/v) goat serum, 0.3% (v/v) Triton X-100 in PBS for guinea-pig anti-AVP (1:16,000; Peninsula Laboratories/Bachem, Belmont, CA, United States) and one of the following: 1) rabbit anti-c-FOS (1:1000, Santa Cruz Biotechnology, Santa Cruz, CA, United States), 2) rabbit anti-PER1 (1:5000), or 3) rabbit anti-PER2 (1:5000). The antibodies for PER1 and PER2 used in this study have been previously characterized and validated (LeSauter et al., 2012). After being rinsed in PBS (3 × 10 min washes), sections were incubated with secondary antibodies Alexa-Fluor^®^ 568 goat anti-rabbit IgG (1:1000, Invitrogen, Carlsbad, CA, United States) and Alexa-Fluor^®^ 488 goat anti-guinea pig IgG (1:1000, Invitrogen) for 2 h at room temperature in the dark. All fluorescence-stained tissue sections were washed in PBS (3 × 10 min washes), mounted on SuperFrost Plus slides (ThermoFisher Scientific, Pittsburgh, PA, United States), dried, and cover-slipped with ProLong^®^ Gold antifade reagent with DAPI (Invitrogen). Serial tissue sections were double-stained with AVP, a canonical marker of the shell or dorsomedial SCN, in order to delineate the SCN core and shell subdivisions (Figure 3A). In each IHC run, sections from each spectral condition were processed together to minimize variability due to handling conditions.

Serial sections throughout the SCN were imaged on a confocal laser-scanning microscope (ZeissLSM700, Carl Zeiss Microscopy, Jena, Germany). A 20 × /0.8NA objective lens was used to provide a lateral resolution of 0.36 μm × 0.36 μm over a 500 μm × 500 μm field-of-view. A z-stack of five images was collected at 3 μm depth intervals with DAPI, Alexa-Fluor^®^ 488 and Alexa-Fluor^®^ 568 imaged sequentially using lasers at 405, 488, and 555 nm, respectively. Image stacks were captured of the right unilateral SCN for each coronal serial image set.

#### *In situ* Hybridization and Co-immunohistochemistry

RNA *in situ* hybridization for *Per1* and *Per2* mRNAs in free-floating brain sections was performed using the RNAscope HybEZ Hybridization System (Advanced Cell Diagnostics Inc., Newark, CA, United States) as previously described (Grabinski et al., 2015). Briefly, a representative mid-rostrocaudal section (between sections with the most discernable core and shell region) was used for each probe. Brain sections were removed from cryoprotectant, washed four times in tris-buffered saline (TBS) for 10 min each, and incubated in Pretreatment 1 at room temperature for 1 h. Sections were washed in 0.5 × TBS (2 × 1 min washes) and distilled water (2 × 1 min washes) before being transferred onto Ultrastick Slides (Andwin Scientific, Canoga Park, CA, United States) and dried at 60°C overnight. Target retrieval (Pretreatment 2), protease pretreatment (Pretreament 3), probe hybridization, and amplification followed by 3,3′-diaminobenzidine (DAB) detection (RNAscope 2.5 HD Detection kit, Advanced Cell Diagnostics Inc.) were performed as previously described (Grabinski et al., 2015). Each RNAscope target probe contained a mixture of 20 ZZ oligonucleotide probes that were bound to target RNA for Per2 (GenBank accession number NM_031678.1; target nucleotide region, 3647 – 4603) or Per1 (GenBank accession number NM_001034125.1; target nucleotide region: 139 – 1112). Following *in situ* hybridization, sections were incubated in 1% hydrogen peroxide for 10 min, washed in TBS (4 × 4 min washes), incubated in 10% (v/v) normal goat serum blocking buffer for 30 min and incubated overnight in guinea-pig anti-AVP (1:2000) in 5% (v/v) goat serum in TBS, all at room temperature. The following day, sections were washed in TBS (4 × 4 min washes) and incubated with biotinylated goat anti-guinea pig secondary antibody (1:500, Vector Laboratories, Burlington, ON, Canada) for 2 h at room temperature. After washing in TBS (4 × 4 min washes), sections were incubated for 2 h in avidin-biotin-peroxidase complex (ABC Elite kit, PK-6100, Vector Laboratories), washed again in TBS (4 × 4 min washes), and the IHC signal was detected using VectorSG peroxidase substrate (SK-4700, Vector Laboratories) for ∼10 min. Once washed in TBS (4 × 1 min washes), slides were dehydrated through a graded series of alcohol (50, 70, 95, 100% ethanol × 1 min each) and cleared in xylene for 2 min before cover-slipping with Cytoseal-60 mounting media (Fisher Scientific, Ottawa, ON, Canada). In a single ISH run, sections from each spectral condition and positive (rat β-actin probe, ACD#409051) and negative (bacterial gene DapB probe, ACD#310043) controls were processed together. Slide images were digitized at 20X using NanoZoomer 2.0RS scanner system (Hamamatsu Photonics, Hamamatsu, Japan). A 10X image of the bilateral SCN for each tissue was extracted for further analyses.

#### Image Quantification

All staining quantification was performed using ImageJ software (version 1.46r, National Institutes of Health, Bethesda, MD, United States). The AVP-expressing region was used to manually outline the SCN boundary and core subregion, while all other measures were automated using ImageJ’s macro programing features. For fluorescence images, Z-stacks were collapsed into maximum-intensity projections, a Gaussian smoothing filter was performed, and the average intensity of the background region was subtracted. For bright-field images, color deconvolution was performed as previously described (Ruifrok and Johnston, 2001) to separate DAB (brown) and Vector SG (blue-gray) signals using vectors derived from positive and negative control tissue. Thresholds were set constant for protein probes and all ISH probes within each batch, and ImageJ’s measure function was used to derive both the area and mean intensity of positive staining for each region of interest. The percentage of the positive area relative to the total area and the mean intensity of the region of interest were calculated. For fluorescent IHC, measures were averaged across all four serial unilateral SCN images for each animal to quantify immunoreactivity in the whole SCN. For the core and shell measurements independently, three out of the four serial sections contained a defined core and shell region that were averaged for SCN subregion quantification. For bright-field images, measures were averaged across the bilateral SCN, including the shell and core independently, for each of the mid-rostrocaudal sections.

### Statistical Analysis

Behavioral, IHC and ISH results are reported as mean ± SEM and analyzed using a one- or two-way ANOVA or a Student’s *t* test (between two groups), as appropriate. For non-normally distributed IHC data, a logarithmic or square root transformation was applied, as appropriate, before proceeding with planned parametric statistical analyses. If a significant main or interaction effect was observed, data were further subjected to Student-Newman-Keuls *post hoc* test when comparing between groups. One-way ANOVA on Ranks with Dunn’s *post hoc* test was performed for behavioral data comparing exposure conditions to pooled dark/handling controls due to unequal variance, as indicated. Statistical significance was set at *p* < 0.05. Statistical analyses and graphical representations were performed using Sigmaplot v13.0 (Systat Software Inc., San Jose, CA, United States).

## Results

### Removing Short Wavelengths From White Light Attenuates Circadian Phase Shifting in an Irradiance and Duration Dependent Manner

**FIGURE 2:**
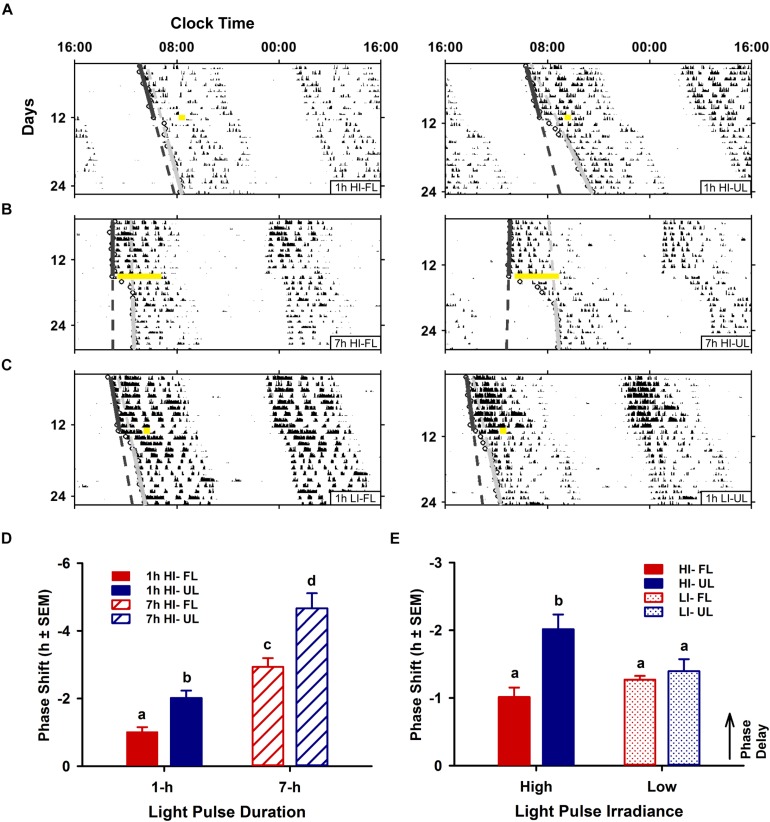
Effects of duration and irradiance on behavioral phase shifts in response to short-wavelength-filtered light (FL) or unfiltered light (UL) exposure. Representative double-plotted actograms of wheel-running activity from free-running animals exposed to a 1-h high irradiance (HI ∼100 μW/cm^2^; **A**) or low irradiance (LI ∼10 μW/cm^2^; **C**) light pulse of completely filtered (< 500 nm, FL; left) or unfiltered light (UL; right) at CT16, and free-running animals exposed to a 7-h HI light pulse **(B)** of FL (left) or UL (right) at CT 13. Phase shifts were assessed by generating regression lines through the activity onsets for at least 10 days before and after photic intervention (indicated by a yellow bar). Completely filtering short wavelengths reduced phase delays for both shorter 1-h and longer 7-h HI light exposures **(D)**, as compared to unfiltered light (1-h HI: FL *n* = 10, UL *n* = 11; 7-h HI: FL *n* = 9, UL *n* = 9). Conversely, filtering visual short wavelengths under a 1-h low irradiance light exposure did not alter phase shifts **(E)**, as compared to unfiltered light (1-h LI: FL *n* = 9, UL *n* = 9). Quantitation of photic phase shifts are reported as group mean ± SEM. Different letters indicate significant differences (*p* < 0.05) after a two-way (exposure condition × irradiance or exposure condition × duration, as indicated) ANOVA with Student-Newman-Keuls *post hoc* test.

Phase shifts for high-irradiance (1 and 7 h) and low-irradiance UL exposure were significantly different than the pooled dark/handling control group (−0.03 ± 0.11; *p* < 0.001). Likewise, high- and low-irradiance FL exposure conditions were also significantly different to the dark control group (*p* < 0.05). Phase shifts were modulated by both exposure condition (filtered vs. unfiltered; *p* < 0.001) and duration (1 h vs 7 h; *p* < 0.001). In the 1-h exposure condition at CT 16, phase shifts were significantly reduced by ∼50% in the < 500 nm filtered light (FL: -1.01 ± 0.14 h; *p* = 0.012) compared to the unfiltered light exposure group (UL: −2.02 ± 0.22 h) (Figures 2A,D). A similar reduction of ∼40% was observed under the 7 h light pulse at CT13, comparing FL (-2.94 ± 0.26; *p* < 0.001) to the UL exposure (-4.67 ± 0.45) (Figures 2B,D). The attenuation in phase resetting by filtered light was not differentially altered based on the duration of exposure [interaction (spectral condition × duration of exposure) *p* = 0.198; Figure 2D].

In contrast to duration, irradiance significantly affected the attenuation in phase resetting [interaction (spectral condition × irradiance) effect *p* = 0.013; Figure 2E]. Unlike the attenuation of phase resetting under high-irradiance 1 h FL compared to UL condition, there was no difference in the magnitude of phase shifts between FL and UL under 1 h low-irradiance conditions (Figures 2C,E).

### Removing Short Wavelengths Form White Light Attenuates Central Pacemaker Activation

**FIGURE 3:**
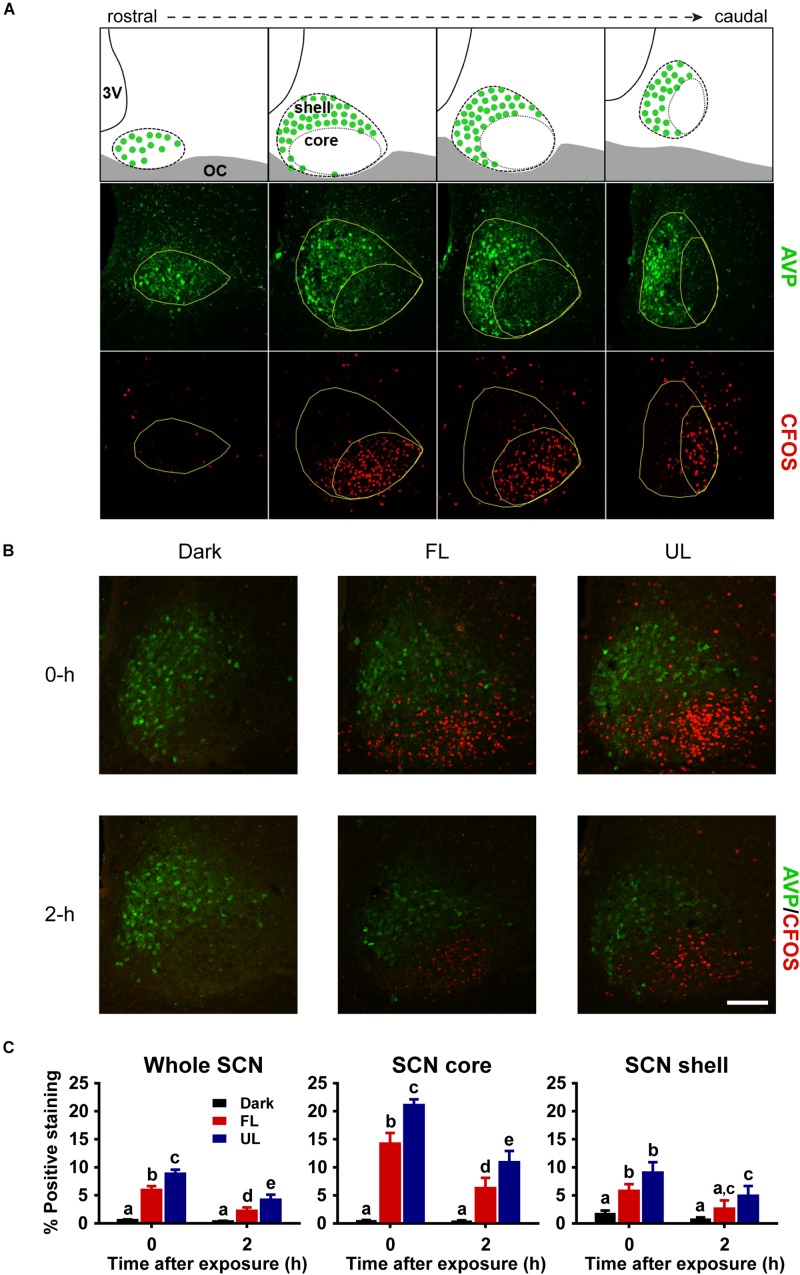
Spatial and temporal effects of filtering short wavelengths on c-FOS immunoreactivity in the SCN. Representative rostral to caudal SCN coronal sections demonstrating core and shell subdivisions, as delineated by AVP-expressing shell region (**A**; Top panel adapted from Moore et al., 2002). 3V: third ventricle; OC: optic chiasm. Representative photomicrographs of mid rostral-caudal coronal SCN sections double-labeled for AVP (green) and c-FOS (red) from animals sacrificed 0-h and 2-h after a high irradiance FL or UL, or dark (control) 1-h exposure at CT16. Scale bar = 75 μm **(B)**. Quantitative analysis examining the proportion of positive area of c-FOS immunoreactivity **(C)** in the whole SCN (left panel), as well as the core (middle panel) and shell (right panel) subdivisions independently. The induction of c-FOS at 0 and 2-h was significantly reduced in the SCN and in the retino-receptive core following FL compare to UL. Data are presented as the group mean ± SEM for each exposure condition at 0-h (*n* = 5) and 2-h (*n* = 6) following light or dark exposure. Different letters indicate significant differences (*p* < 0.05) after a two-way (exposure condition × time after exposure) ANOVA with Student-Newman-Keuls *post hoc* test.

The robust induction of c-FOS, a reliable marker of SCN activation, detected in the whole SCN and in the core subdivision immediately following UL exposure was decreased ∼33% in the FL group (FL vs. UL 0 h, whole SCN *p* = 0.002; SCN core *p* = 0.001; Figures 3B,C). As expected, light-exposure induced c-FOS expression was reduced 2 h after the light exposure (effect of time *p* < 0.01). The attenuation of c-FOS induction under the FL compared to the UL condition persisted 2 h after the end of the light pulses with similar decreases of ∼46 and ∼39% in the whole SCN and core, respectively (FL vs. UL 2 h, whole SCN: *p* = 0.039; SCN core *p* = 0.035; Figure 3C). While the highest levels of c-FOS expression were observed in the retino-receptive SCN core (Figure 3C middle panel), there were also UL-induced increases in the shell region 0 h (*p* < 0.0001) and 2 h (*p* = 0.016; Figure 3C right panel). Moreover, this increase in c-FOS expression in the shell subdivision was reduced by filtering blue light (FL compared to UL exposure groups, *p* = 0.042), although *post hoc* comparisons did not reveal individual significant differences at either time point after exposure (FL vs. UL 0 h and 2 h *p* > 0.05).

### Removing Short Wavelengths Form White Light Attenuates Changes in the Molecular-Clock Mechanism

#### Changes in Light-Induced *Per1* and *Per2* Gene Expression

**FIGURE 4:**
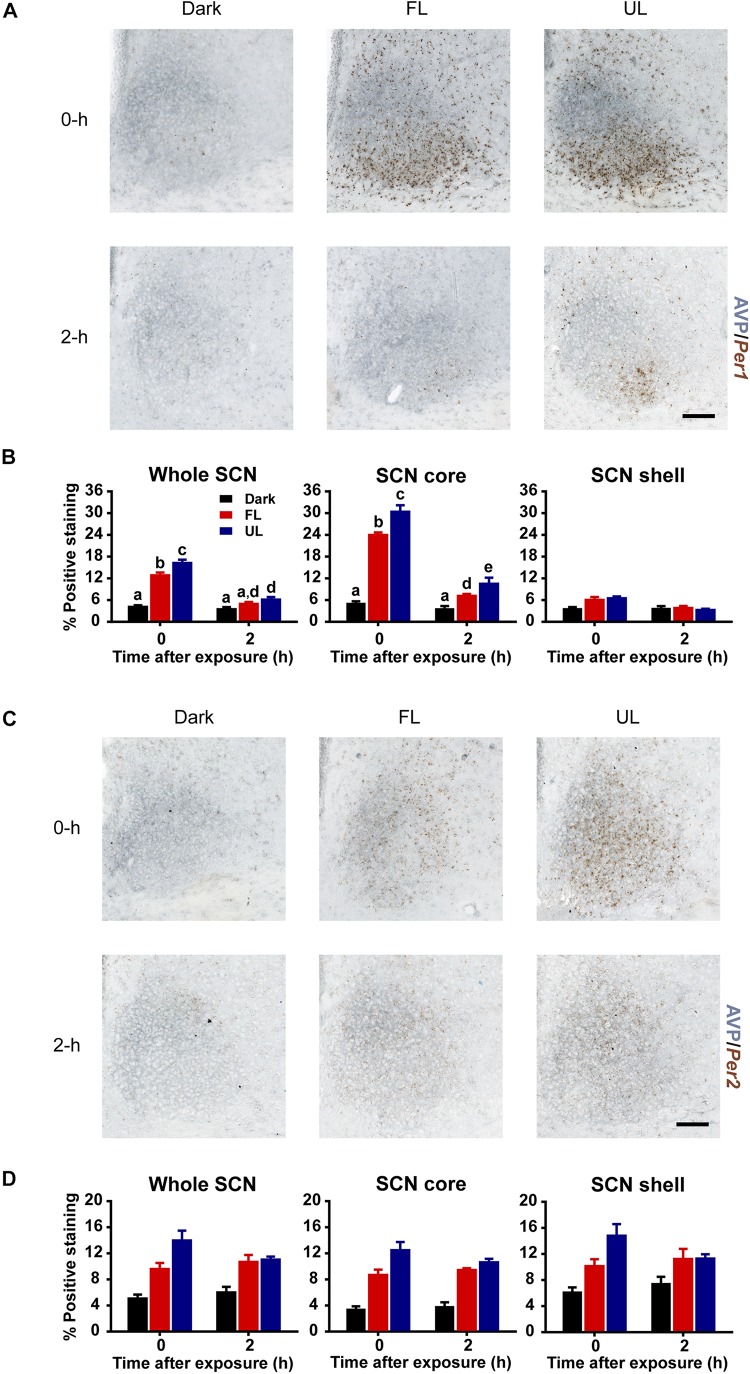
Spatial and temporal effects of filtering short wavelengths on *Per1* and *Per2* mRNA expression in the SCN. Representative photomicrographs of mid rostral-caudal coronal SCN sections double-labeled for AVP (blue-gray) and *Per1* or *Per2* mRNA (brown) from animals sacrificed 0-h and 2-h after a high irradiance FL or UL, or dark (control) 1-h exposure at CT16. Scale bar = 100 μm **(A,C)**. Quantitative analysis examining the proportion of positively stained area of *Per1* **(B)** or *Per2* **(D)** gene expression in the entire SCN (left panel), as well as the core (middle panel) and shell (right panel) subdivisions independently. The induction of *Per1* mRNA at 0-h and 2-h was significantly reduced in the in the retino-receptive core following FL compared to UL exposure **(B)**. Data are presented as the group mean ± SEM for each exposure condition at 0-h (*n* = 5) and 2-h (*n* = 5) following light or dark exposure. Different letters indicate significant differences (*p* < 0.05) after a two-way (exposure condition × time after exposure) ANOVA with Student-Newman-Keuls *post hoc* test.

Upregulation of *Per1* mRNA following an unfiltered light pulse was highest in the core region (Figures 4A,B), in agreement with previous reports (Yan et al., 1999; Yan and Okamura, 2002). There was a ∼ 22% decrease in *Per1* expression in the whole SCN, and the core subdivision, immediately following FL compared to UL exposure (FL vs. UL 0 h, whole SCN *p* = 0.002; SCN core *p* = < 0.001; Figure 4B). Comparable reductions of ∼25% were observed in *Per1* expression levels in the whole SCN and core subdivision 2 h following FL compared to UL exposure (*p* = 0.049), with the overall expression levels decreasing over time (*p* < 0.001). There may be marginal reductions of *Per1* expression in the shell region, particularly immediately following light exposure; however, this did not reach statistical significance (*p* = 0.053).

Similar to *Per1* expression levels, light induction of *Per2* mRNA was observed in the SCN (Figures 4C,D). *Per2* mRNA upregulation was distributed more uniformly throughout the SCN, including both core and shell subdivisions (effect of condition in the whole SCN, core and shell *p* < 0.001). Moreover, the *Per2* upregulation was significantly less in FL compared to UL light exposure in the whole SCN and core region but was not in the shell region (whole SCN *p* = 0.022, SCN core *p* = 0.002, SCN shell *p* = 0.063; Figure 4D). However, there was no effect of time on *Per2* expression in whole SCN, core, or shell (*p* > 0.05), since light-induced *Per2* expression remained high even 2 h following light exposure. This sustained upregulation in *Per2* expression is similar to previous observations (Yan et al., 1999). Since the effect of time was not significant, nor the interaction of condition and time, data for each condition was pooled across timepoints. Combining both timepoints showed reductions of ∼20% in Per2 expression in the whole SCN (*p* = 0.027) and core (*p* = 0.002), but not the shell subdivision (*p* = 0.072), following FL compared to UL exposure.

#### Changes in Light-Induced PER1 Protein Expression

**FIGURE 5:**
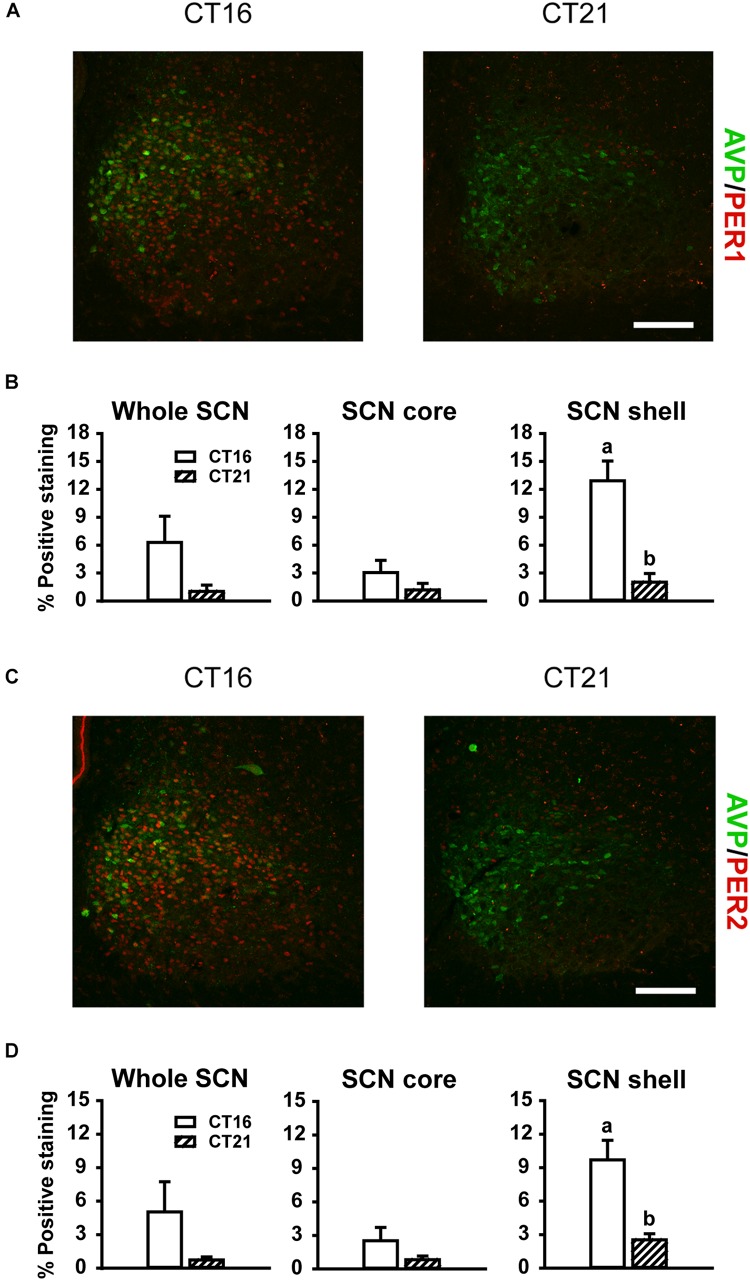
Representative photomicrographs of mid rostral-caudal coronal SCN sections double-labeled for AVP (green) and PER1 or PER2 (red) from free-running animals sacrificed during the first (CT 16; *n* = 3) or second (CT 21; *n* = 3) half of the subjective night. Scale bar = 75 μm **(A,C)**. Quantitative analysis examining the proportion of positively stained area of PER1 **(B)** or PER2 **(D)** immunoreactivity in the entire SCN (left panel), as well as the core (middle panel) and shell (right panel) subdivisions independently. Endogenous PER1 and PER2 immunoreactivity was observed in the shell region during the earlier half of the subjective night at CT 16, as compared to the CT 21. Data are presented as the mean ± SEM. Different letters indicate significant differences (*p* < 0.05) after a Student’s *t* test.

**FIGURE 6:**
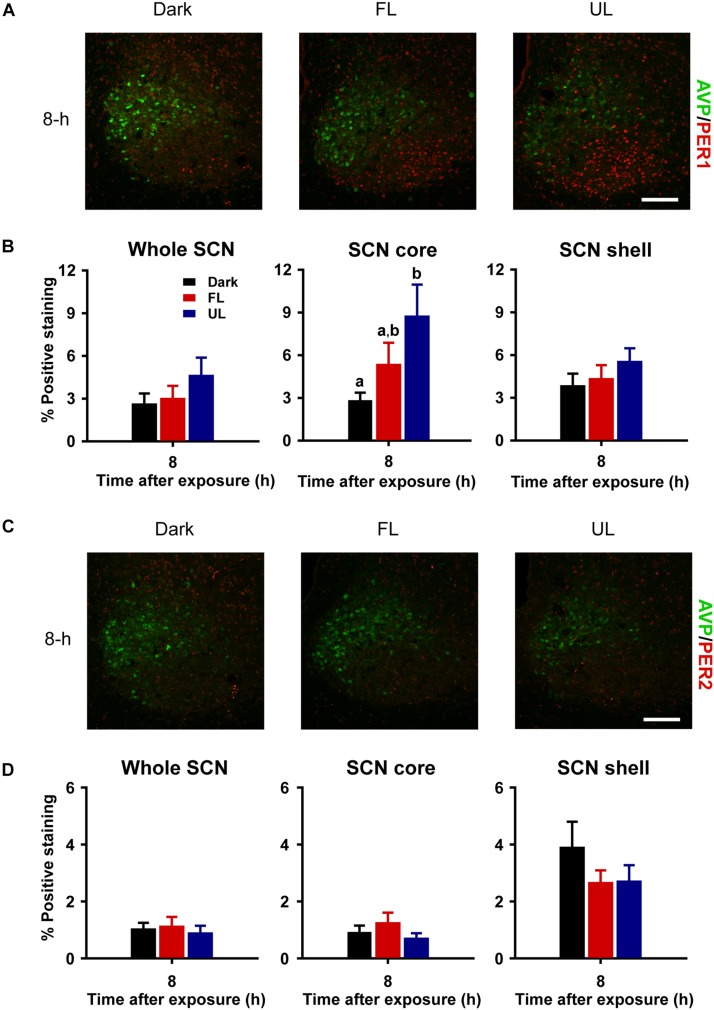
Delayed and regionally specific upregulation of photic-induced PER1 immunoreactivity in the SCN. Representative photomicrographs of mid rostral-caudal coronal SCN sections double-labeled for AVP (green) and PER1 or PER2 (red) from animals sacrificed 8-h after high irradiance FL or UL, or dark (control) 1-h exposures at CT16. Scale bar = 75 μm **(A,C)**. Quantitative analysis examining the proportion of positively stained area of PER1 **(B)** or PER2 **(D)** immunoreactivity in the entire SCN (left panel), as well as the core (middle panel) and shell (right panel) subdivisions independently. The induction of PER1-IR was observed in the core region under the UF condition only, as compared to the dark control **(B)**. Data are presented as the mean ± SEM at 8-h (dark *n* = 6, FL *n* = 7, UL *n* = 7) per exposure condition. Different letters indicate significant differences (*p* < 0.05) after a one-way (exposure condition) ANOVA with Student-Newman-Keuls *post hoc* test.

Consistent with previous reports (Field et al., 2000; Yan and Silver, 2004), robust expression of PER1 and PER2 protein was observed only in the shell region of the SCN from animals maintained under free-running DD conditions during the early as compared to late subjective night (CT 16 vs CT 21; PER1 *p* = 0.009, Figures 5A,B right panel; PER2 *p* = 0.018, Figures 5C,D right panel). Due to a substantial ∼4–9 h delay in light-evoked PER1 and PER2 protein levels previously reported (Field et al., 2000; Harmar et al., 2002; von Gall et al., 2003; Yan and Silver, 2004), brain tissue was collected 8 h following FL, UL or dark exposure. Compared to expression levels under the dark-control condition, there was a significant increase in PER1 expression in the SCN core region only (*p* = 0.029) following light exposure (Figures 6A,B). While UL exposure increased PER1 expression in the SCN core (*p* = 0.023), FL exposure did not appear to induce a statistically significant increase in PER1 expression compared to the dark-control condition (*p* = 0.147; Figure 6B middle panel). PER2 expression was unaffected by photic stimuli and all exposure groups had low basal levels of PER2 expression (*p* > 0.05, Figures 6C,D).

## Discussion

Non-visual responses to ocular light exposure including changes in hormone secretion and phase shifts in locomotor activity rhythms have a peak sensitivity to short-wavelength (blue) visible light and are mediated via ipRGCs primarily through the photopigment melanopsin (Takahashi et al., 1984; Boulos, 1995; Brainard et al., 2001; Thapan et al., 2001; Lockley et al., 2003, 2006; Cajochen et al., 2005; Bedrosian et al., 2013). Reducing the short-wavelength content from polychromatic white light can attenuate the acute changes in hormone secretion and gene expression (Kayumov et al., 2005; Sasseville et al., 2006; Rahman et al., 2008; Sasseville and Hebert, 2010; Rahman et al., 2011, 2013; Gil-Lozano et al., 2016), but the effects on phase resetting responses are not known. We show, for the first time, that filtering visual short wavelengths from high irradiance polychromatic light at night can attenuate circadian phase resetting and alterations in associated markers of SCN activation. These results suggest that depleting the blue content of polychromatic light may be an effective method of attenuating direct and pacemaker mediated phase resetting when these effects are not desirable. Importantly, the efficacy of this intervention was intensity dependent, such that at a low-irradiance, depleting the blue content of polychromatic light did not reduce behavioral phase shifts as compared to unfiltered light exposure. These results provide novel insight on the photobiologic responses to short-wavelength depleted light and have important implications for designing lighting interventions.

In the current study, filtering short wavelengths < 500 nm from high-irradiance polychromatic white light at night reduced phase shifts in locomotor activity by ∼40–50% [range of effect size (Cohen’s *d*) 1.58–1.68] compared to unfiltered polychromatic white light. These results are similar to the reduction in locomotor activity phase shifts observed in melanopsin knockout mice (Panda et al., 2002; Ruby et al., 2002) suggesting that the modulation of the melanopic signaling pathway likely explains the attenuation in phase shifts observed in this study. This is further supported by the ∼75% reduction in estimated melanopsin activation under the FL compared to UL condition, as assessed using melanopic response-weighted irradiance functions (Figures 1C,D; Lucas et al., 2014). Given that melanopsin knockout mice still retain nearly half of their light-evoked phase resetting response (Panda et al., 2002; Ruby et al., 2002), this also underscores key contributions from rod and cone photoreceptors. Consistent with this idea, we observed a significant reduction, but not complete abolishment of photic circadian phase resetting following FL exposure. This residual phase resetting under the FL condition may be, at least in part, due to the activation of classical photoreceptors; however, this warrants further investigation. Additionally, these results suggest that chronic exposure to even filtered light may cause substantial phase resetting and repeated phase resetting, albeit of smaller magnitude, which may promote circadian disruption and adverse health effects associated with circadian disruption. Future studies are required to assess the impact of chronic filtered light exposure at night on health outcomes, however.

The involvement of the rod and cone visual photoreceptors in mediating some part of the total phase resetting effects of light exposure may also explain the lack of attenuation of phase resetting by filtering short wavelengths at low irradiances. Results from rodent and human studies show more robust contributions of classical photoreceptors in phase resetting responses in low light irradiances as compared to high irradiance conditions (Dkhissi-Benyahya et al., 2007; Altimus et al., 2010; Gooley et al., 2010). This suggests that filtering short wavelengths under low irradiance conditions does not reduce the activation of visual photoreceptors, which can continue to signal to the pacemaker and contribute to phase resetting. This finding is particularly important because reducing the blue content by either filtering short wavelengths (Sasseville et al., 2006; Burkhart and Phelps, 2009; Figueiro et al., 2011; Santhi et al., 2012; Chellappa et al., 2013; van der Lely et al., 2015) or using blue-depleted light sources [e.g., spectrally tuned LEDs (Santhi et al., 2012)] may be used as possible countermeasures for pre-bedtime/evening light exposure. Such an intervention is usually combined with reducing the intensity of light exposure. Our results suggest that the coupled intervention may not be optimal for attenuating unwanted circadian phase resetting induced by light exposure. Additional studies are needed to determine whether blue depletion of indoor bedroom-intensity lighting effectively attenuates the disruptive effects of nocturnal light exposure on sleep and health outcomes (Chang et al., 2015; Gronli et al., 2016). An additional important consideration for using spectral tuning to attenuate the disruptive effects of light exposure at night is the impact on visual performance. We have previously shown impaired color discrimination caused by blue-depleted light (Rahman et al., 2017), although novel technology using metamerism can selectively attenuate NIF responses without altering photometric (color and luminance) characteristics (Allen et al., 2018).

Since the 1 and 7-h high irradiance filtered-light-exposure conditions had similar reductions (∼40–50%) in phase resetting, we chose the shorter 1-h light pulse to assess the interaction between intensity and spectrum to minimize saturating the phase resetting response, which could otherwise prevent observing any potential interactions. Given our findings, future studies are required to investigate a possible three-way interaction between spectrum, intensity and duration. Additionally, future studies are needed to assess whether the underlying neural and molecular responses differ between low- and high-intensity spectrally tuned lighting. Recent studies suggest that divergent neural pathways exist for NIF responses (Rupp et al., 2019), and phase resetting response mechanisms may differ based on the duration of the stimulus (Najjar and Zeitzer, 2016; Kaladchibachi et al., 2019; Kronauer et al., 2019), which may also be the case for intensity.

Previous work has shown that ∼6-h light pulses generate the largest magnitude of phase delays (Comas et al., 2006). Moreover, photoreceptor contribution to phase resetting changes dynamically with exposure duration (Gooley et al., 2010). The relative contribution of cones and ipRGCs are similar at the start of a long duration continuous light exposure but start to differ significantly ∼1.6 h after the onset of the light exposure. Results from a short duration (∼1 h) stimulus cannot always be extended to a long duration stimulus (∼7 h); therefore, we also tested the phase resetting response to filtered and unfiltered lighting in response to a 7 h light pulse. We observed comparable ∼40–50% reductions in phase resetting following the high-irradiance FL condition compared to the UL condition using both a 1-h and 7-h light pulse duration. This suggests that the efficacy of blue depletion in attenuating phase shifts is preserved under long saturating photic stimuli.

Our study shows that filtering short wavelengths modulates phase resetting via the canonical molecular pathway that mediates light-induced phase shifts. Temporal and spatial expression patterns of c-FOS, a reliable marker of light exposure-induced SCN activation, showed a significant reduction in SCN activation in response to high-irradiance FL compared to UL exposure. As expected, c-FOS induction was most prominent in the retino-receptive core region, with significant decreases at 0 h and 2 h following FL in both the proportion of c-FOS immunoreactivity as compared to UL exposure. Similar reductions of c-FOS expression levels were reported in melanopsin deficient mice, displaying a ∼50% reduction in c-FOS immunoreactivity immediately following a high irradiance 1-h light pulse (Tsai et al., 2009).

It is well established that the magnitude of photic phase resetting is correlated with increases in *Per1* and *Per2* expression levels in the SCN (Akiyama et al., 1999; Wakamatsu et al., 2001; Tischkau et al., 2003). A recent study showed greater expression of *Per1*, *Per2*, and *Fos* mRNA in the SCN following a 1-h exposure of monochromatic blue light (λ_*max*_ 470 nm) compared to green light (λ_*max*_ 530 nm), and that this blue light induction was melanopsin-dependent (Pilorz et al., 2016). This is consistent with the reduction in *Per1* and *Per2* gene expression in the SCN observed in the current study following high-irradiance FL compared to UL exposure. In response to a phase-delaying light pulse, *Per1* upregulation was localized within the SCN core region, whereas *Per2* mRNA was distributed more uniformly throughout the SCN core and shell, as previously reported (Yan and Okamura, 2002).

Both PER1 and PER2 proteins exhibit similar circadian oscillations, reaching peak levels during the early subjective night in the dorsomedial shell region and gradually falling to nadir levels in the late subjective night to early subjective day, as reported previously (Field et al., 2000; Yan and Silver, 2004) and observed in this study (Figure 5). Additionally, PER1 protein levels increase in the SCN ∼4–9 h following a light pulse administered during the subjective night (Harmar et al., 2002; von Gall et al., 2003; Yan and Silver, 2004); however, conflicting results have been reported for PER2, including minimal to increased changes in expressive levels (Field et al., 2000; Harmar et al., 2002; Yan and Silver, 2004). This suggests that only light-exposure induced increases in PER1 are essential in phase resetting. We found light-evoked increases of PER1 expression in the retino-receptive core 8 h following high-irradiance UL exposure, but not under FL exposure. Considering the substantial variability in the delay of light evoked PER1 induction (∼4–9-h) in the SCN, it is possible that filtered and unfiltered light exposure have differential dynamics in PER1 protein induction which warrants further investigation to assess this response dynamic. Moreover, low-irradiance filtered light did not reduce behavioral phase shifts, however, future studies are needed to explore photic-induced molecular responses in the SCN under low-irradiance conditions.

Running-wheel activity is a reliable and widely used measure of circadian phase in rodent studies, although there are many behavioral and physiological measures that exhibit daily rhythms. The SCN uses different signaling pathways for locomotor and endocrine rhythms (Meyer-Bernstein et al., 1999), and the use of filtered and unfiltered lighting may differentially affect these measures. Thus, it would be interesting to associate our findings with endocrine outcome measures in future studies. While the rat provides an excellent model to examine the mechanistic aspects of spectral tuning, caution must be exercised in translating our findings to humans. While our findings suggest an effective countermeasure for evening and/or nocturnal light exposure on human sleep and circadian physiology, the effects of blue-depleted light on phase advances needs to be assessed in humans. The phase response function in humans is asymmetric with larger phase delays than phase advances (Khalsa et al., 2003; St Hilaire et al., 2012; Ruger et al., 2013) and both phase advances and delays are critical for designing an effective tool for optimizing circadian adaptation. Nevertheless, our findings provide needed insight on the acute and phase resetting photobiologic responses of blue depleted polychromatic light.

In summary, we demonstrate that filtering visual short wavelengths from high irradiance polychromatic light reduces circadian phase resetting. Furthermore, we show that this corresponds to regionally specific decreases in photic-induced molecular responses in the SCN, including c-FOS and core clock expression. This spectral tuning did not have the same efficacy on phase resetting of circadian behavioral rhythms under low irradiance levels. In a broader context, these findings should help direct future lighting designs to consider both wavelength and irradiance manipulations when employing spectral optimization to minimize circadian disruption in response to light at night.

## Data Availability

The datasets generated for this study are available on request to the corresponding author.

## Ethics Statement

The animal study was reviewed and approved by the University of Toronto Animal Care Committee in accordance with federal guidelines (Canadian Council on Animal Care).

## Author Contributions

BG, CS, TB, MR, RC, and SR conceived and designed the experiments. BG collected the data. BG, JJ, MR, and SR analyzed and visualized the data. BG and SR wrote the manuscript. BG, JJ, CS, TB, MR, RC, and SR reviewed and edited the manuscript. RC and SR acquired funding and administered the project. MR, RC, and SR provided the resources.

## Conflict of Interest Statement

RC is a scientific advisory member for Allergan, Bayer, EMD-Serono, Ferring, Merck, and OvaScience. RC serves as the Medical Director for Insception-Lifebank Cord Blood Bank. RC serves as the Scientific Director for TRIO Fertility. RC owns equity in Circadian-Zirclight, OvaScience and Fertilify. RC has received royalties from Teva and Up-to-Date. RC has provided paid consulting services to Fertility Nutraceuticals. SR holds patents for prevention of circadian rhythm disruption by using optical filters and improving sleep performance in subjects exposed to light at night. SR owns equity in Melcort Inc. SR has provided paid consulting services to Sultan and Knight Limited, Bambu Vault LLC. SR has received honoraria as an invited speaker and travel funds from Starry Skies Lake Superior, University of Minnesota Medical School, PennWell Corp., Seoul Semiconductor Co., LTD. SR’s interests were reviewed and managed by the Brigham and Women’s Hospital and Partners HealthCare in accordance with their conflict of interest policies. The remaining authors declare that the research was conducted in the absence of any commercial or financial relationships that could be construed as a potential conflict of interest.

## References

[B1] Akiyama M. Kouzu Y. Takahashi S. Wakamatsu H. Moriya T. Maetani M. (1999). Inhibition of light- or glutamate-induced mPer1 expression represses the phase shifts into the mouse circadian locomotor and suprachiasmatic firing rhythms. *J. Neurosci.* 19 1115–1121. 10.1523/jneurosci.19-03-01115.1999 9920673 PMC6782139

[B2] Allen A. E. Hazelhoff E. M. Martial F. P. Cajochen C. Lucas R. J. (2018). Exploiting metamerism to regulate the impact of a visual display on alertness and melatonin suppression independent of visual appearance. *Sleep* 41:zsy100. 10.1093/sleep/zsy100 29788219 PMC6093320

[B3] Altimus C. M. Guler A. D. Alam N. M. Arman A. C. Prusky G. T. Sampath A. P. (2010). Rod photoreceptors drive circadian photoentrainment across a wide range of light intensities. *Nat. Neurosci.* 13 1107–1112. 10.1038/nn.2617 20711184 PMC2928860

[B4] Bedrosian T. A. Vaughn C. A. Galan A. Daye G. Weil Z. M. Nelson R. J. (2013). Nocturnal light exposure impairs affective responses in a wavelength-dependent manner. *J. Neurosci.* 33 13081–13087. 10.1523/JNEUROSCI.5734-12.2013 23926261 PMC6619722

[B5] Boivin D. B. Boudreau P. James F. O. Kin N. M. (2012). Photic resetting in night-shift work: impact on nurses’ sleep. *Chronobiol. Int.* 29 619–628. 10.3109/07420528.2012.675257 22621359

[B6] Boivin D. B. James F. O. (2002). Circadian adaptation to night-shift work by judicious light and darkness exposure. *J. Biol. Rhythms* 17 556–567. 10.1177/0748730402238238 12465889

[B7] Boulos Z. (1995). Wavelength dependence of light-induced phase shifts and period changes in hamsters. *Physiol. Behav.* 57 1025–1033. 10.1016/0031-9384(95)00015-b 7652020

[B8] Brainard G. C. Hanifin J. P. Greeson J. M. Byrne B. Glickman G. Gerner E. (2001). Action spectrum for melatonin regulation in humans: evidence for a novel circadian photoreceptor. *J. Neurosci.* 21 6405–6412. 10.1523/jneurosci.21-16-06405.2001 11487664 PMC6763155

[B9] Burkhart K. Phelps J. R. (2009). Amber lenses to block blue light and improve sleep: a randomized trial. *Chronobiol. Int.* 26 1602–1612. 10.3109/07420520903523719 20030543

[B10] Cajochen C. Munch M. Kobialka S. Krauchi K. Steiner R. Oelhafen P. (2005). High sensitivity of human melatonin, alertness, thermoregulation, and heart rate to short wavelength light. *J. Clin. Endocrinol. Metab.* 90 1311–1316. 10.1210/jc.2004-0957 15585546

[B11] Chang A. M. Aeschbach D. Duffy J. F. Czeisler C. A. (2015). Evening use of light-emitting eReaders negatively affects sleep, circadian timing, and next-morning alertness. *Proc. Natl. Acad. Sci. U.S.A.* 112 1232–1237. 10.1073/pnas.1418490112 25535358 PMC4313820

[B12] Chellappa S. L. Steiner R. Oelhafen P. Lang D. Gotz T. Krebs J. (2013). Acute exposure to evening blue-enriched light impacts on human sleep. *J. Sleep Res.* 22 573–580. 10.1111/jsr.12050 23509952

[B13] Comas M. Beersma D. G. Spoelstra K. Daan S. (2006). Phase and period responses of the circadian system of mice (Mus musculus) to light stimuli of different duration. *J. Biol. Rhythms* 21 362–372. 10.1177/0748730406292446 16998156

[B14] Czeisler C. A. Gooley J. J. (2007). Sleep and circadian rhythms in humans. *Cold Spring Harb. Symp. Quant. Biol.* 72 579–597. 10.1101/sqb.2007.72.064 18419318

[B15] Czeisler C. A. Johnson M. P. Duffy J. F. Brown E. N. Ronda J. M. Kronauer R. E. (1990). Exposure to bright light and darkness to treat physiologic maladaptation to night work. *N. Engl. J. Med.* 322 1253–1259. 10.1056/nejm199005033221801 2325721

[B16] Dkhissi-Benyahya O. Gronfier C. De Vanssay W. Flamant F. Cooper H. M. (2007). Modeling the role of mid-wavelength cones in circadian responses to light. *Neuron* 53 677–687. 10.1016/j.neuron.2007.02.005 17329208 PMC1950159

[B17] Duffy J. F. Kronauer R. E. Czeisler C. A. (1996). Phase-shifting human circadian rhythms: influence of sleep timing, social contact and light exposure. *J. Physiol.* 495 (Pt 1) 289–297. 10.1113/jphysiol.1996.sp021593 8866371 PMC1160744

[B18] Field M. D. Maywood E. S. O’brien J. A. Weaver D. R. Reppert S. M. Hastings M. H. (2000). Analysis of clock proteins in mouse SCN demonstrates phylogenetic divergence of the circadian clockwork and resetting mechanisms. *Neuron* 25 437–447. 10.1016/s0896-6273(00)80906-x 10719897

[B19] Figueiro M. G. Wood B. Plitnick B. Rea M. S. (2011). The impact of light from computer monitors on melatonin levels in college students. *Neuro Endocrinol. Lett.* 32 158–163. 21552190

[B20] Gil-Lozano M. Hunter P. M. Behan L. A. Gladanac B. Casper R. F. Brubaker P. L. (2016). Short-term sleep deprivation with nocturnal light exposure alters time-dependent glucagon-like peptide-1 and insulin secretion in male volunteers. *Am. J. Physiol. Endocrinol. Metab.* 310 E41–E50. 10.1152/ajpendo.00298.2015 26530153

[B21] Gooley J. J. Rajaratnam S. M. Brainard G. C. Kronauer R. E. Czeisler C. A. Lockley S. W. (2010). Spectral responses of the human circadian system depend on the irradiance and duration of exposure to light. *Sci. Transl. Med.* 2:31ra33. 10.1126/scitranslmed.3000741 20463367 PMC4414925

[B22] Grabinski T. M. Kneynsberg A. Manfredsson F. P. Kanaan N. M. (2015). A method for combining RNAscope in situ hybridization with immunohistochemistry in thick free-floating brain sections and primary neuronal cultures. *PLoS One* 10:e0120120. 10.1371/journal.pone.0120120 25794171 PMC4368734

[B23] Gronli J. Byrkjedal I. K. Bjorvatn B. Nodtvedt O. Hamre B. Pallesen S. (2016). Reading from an iPad or from a book in bed: the impact on human sleep. A randomized controlled crossover trial. *Sleep Med.* 21 86–92. 10.1016/j.sleep.2016.02.006 27448477

[B24] Guler A. D. Ecker J. L. Lall G. S. Haq S. Altimus C. M. Liao H. W. (2008). Melanopsin cells are the principal conduits for rod-cone input to non-image-forming vision. *Nature* 453 102–105. 10.1038/nature06829 18432195 PMC2871301

[B25] Harmar A. J. Marston H. M. Shen S. Spratt C. West K. M. Sheward W. J. (2002). The VPAC(2) receptor is essential for circadian function in the mouse suprachiasmatic nuclei. *Cell* 109 497–508. 10.1016/s0092-8674(02)00736-5 12086606

[B26] Hickie I. B. Rogers N. L. (2011). Novel melatonin-based therapies: potential advances in the treatment of major depression. *Lancet* 378 621–631. 10.1016/S0140-6736(11)60095-0 21596429

[B27] James F. O. Cermakian N. Boivin D. B. (2007). Circadian rhythms of melatonin, cortisol, and clock gene expression during simulated night shift work. *Sleep* 30 1427–1436. 10.1093/sleep/30.11.1427 18041477 PMC2082093

[B28] Kaladchibachi S. Negelspach D. C. Zeitzer J. M. Fernandez F. (2019). Optimization of circadian responses with shorter and shorter millisecond flashes. *Biol. Lett.* 15:20190371. 10.1098/rsbl.2019.0371 31387472 PMC6731482

[B29] Kayumov L. Casper R. F. Hawa R. J. Perelman B. Chung S. A. Sokalsky S. (2005). Blocking low-wavelength light prevents nocturnal melatonin suppression with no adverse effect on performance during simulated shift work. *J. Clin. Endocrinol. Metab.* 90 2755–2761. 10.1210/jc.2004-2062 15713707

[B30] Khalsa S. B. Jewett M. E. Cajochen C. Czeisler C. A. (2003). A phase response curve to single bright light pulses in human subjects. *J. Physiol.* 549 945–952. 10.1113/jphysiol.2003.040477 12717008 PMC2342968

[B31] Kiessling S. Sollars P. J. Pickard G. E. (2014). Light stimulates the mouse adrenal through a retinohypothalamic pathway independent of an effect on the clock in the suprachiasmatic nucleus. *PLoS One* 9:e92959. 10.1371/journal.pone.0092959 24658072 PMC3962469

[B32] Kronauer R. E. St Hilaire M. A. Rahman S. A. Czeisler C. A. Klerman E. B. (2019). An exploration of the temporal dynamics of circadian resetting responses to short- and long-duration light exposures: cross-species consistencies and differences. *J Biol. Rhythms* 10.1177/0748730419862702 [Epub ahead of print]. 31368391 PMC7363039

[B33] LeSauter J. Lambert C. M. Robotham M. R. Model Z. Silver R. Weaver D. R. (2012). Antibodies for assessing circadian clock proteins in the rodent suprachiasmatic nucleus. *PLoS One* 7:e35938. 10.1371/journal.pone.0035938 22558277 PMC3338757

[B34] Lockley S. W. Brainard G. C. Czeisler C. A. (2003). High sensitivity of the human circadian melatonin rhythm to resetting by short wavelength light. *J. Clin. Endocrinol. Metab.* 88 4502–4505. 12970330 10.1210/jc.2003-030570

[B35] Lockley S. W. Evans E. E. Scheer F. A. Brainard G. C. Czeisler C. A. Aeschbach D. (2006). Short-wavelength sensitivity for the direct effects of light on alertness, vigilance, and the waking electroencephalogram in humans. *Sleep* 29 161–168. 16494083

[B36] Lucas R. J. Peirson S. N. Berson D. M. Brown T. M. Cooper H. M. Czeisler C. A. (2014). Measuring and using light in the melanopsin age. *Trends Neurosci.* 37 1–9. 10.1016/j.tins.2013.10.004 24287308 PMC4699304

[B37] Meyer-Bernstein E. L. Jetton A. E. Matsumoto S. I. Markuns J. F. Lehman M. N. Bittman E. L. (1999). Effects of suprachiasmatic transplants on circadian rhythms of neuroendocrine function in golden hamsters. *Endocrinology* 140 207–218. 10.1210/en.140.1.207 9886827

[B38] Moore R. Y. Speh J. C. Leak R. K. (2002). Suprachiasmatic nucleus organization. *Cell Tissue Res.* 309 89–98. 12111539 10.1007/s00441-002-0575-2

[B39] Morris C. J. Yang J. N. Garcia J. I. Myers S. Bozzi I. Wang W. (2015). Endogenous circadian system and circadian misalignment impact glucose tolerance via separate mechanisms in humans. *Proc. Natl. Acad. Sci. U.S.A.* 112 E2225–E2234. 10.1073/pnas.1418955112 25870289 PMC4418873

[B40] Najjar R. P. Zeitzer J. M. (2016). Temporal integration of light flashes by the human circadian system. *J. Clin. Invest.* 126 938–947. 10.1172/JCI82306 26854928 PMC4767340

[B41] Panda S. Sato T. K. Castrucci A. M. Rollag M. D. Degrip W. J. Hogenesch J. B. (2002). Melanopsin (Opn4) requirement for normal light-induced circadian phase shifting. *Science* 298 2213–2216. 10.1126/science.1076848 12481141

[B42] Paul M. A. Miller J. C. Love R. J. Lieberman H. Blazeski S. Arendt J. (2009). Timing light treatment for eastward and westward travel preparation. *Chronobiol. Int.* 26 867–890. 10.1080/07420520903044331 19637048

[B43] Pilorz V. Tam S. K. Hughes S. Pothecary C. A. Jagannath A. Hankins M. W. (2016). Melanopsin regulates both sleep-promoting and arousal-promoting responses to light. *PLoS Biol.* 14:e1002482. 10.1371/journal.pbio.1002482 27276063 PMC4898879

[B44] Rahman S. A. Kollara A. Brown T. J. Casper R. F. (2008). Selectively filtering short wavelengths attenuates the disruptive effects of nocturnal light on endocrine and molecular circadian phase markers in rats. *Endocrinology* 149 6125–6135. 10.1210/en.2007-1742 18687787

[B45] Rahman S. A. Marcu S. Shapiro C. M. Brown T. J. Casper R. F. (2011). Spectral modulation attenuates molecular, endocrine, and neurobehavioral disruption induced by nocturnal light exposure. *Am. J. Physiol. Endocrinol. Metab.* 300 E518–E527. 10.1152/ajpendo.00597.2010 21177289

[B46] Rahman S. A. Shapiro C. M. Wang F. Ainlay H. Kazmi S. Brown T. J. (2013). Effects of filtering visual short wavelengths during nocturnal shiftwork on sleep and performance. *Chronobiol. Int.* 30 951–962. 10.3109/07420528.2013.789894 23834705 PMC3786545

[B47] Rahman S. A. St Hilaire M. A. Gronfier C. Chang A. M. Santhi N. Czeisler C. A. (2018). Functional decoupling of melatonin suppression and circadian phase resetting in humans. *J. Physiol.* 596 2147–2157. 10.1113/JP275501 29707782 PMC5983136

[B48] Rahman S. A. St Hilaire M. A. Lockley S. W. (2017). The effects of spectral tuning of evening ambient light on melatonin suppression, alertness and sleep. *Physiol. Behav.* 177 221–229. 10.1016/j.physbeh.2017.05.002 28472667 PMC5536841

[B49] Rajaratnam S. M. Barger L. K. Lockley S. W. Shea S. A. Wang W. Landrigan C. P. (2011). Sleep disorders, health, and safety in police officers. *JAMA* 306 2567–2578. 10.1001/jama.2011.1851 22187276

[B50] Regente J. De Zeeuw J. Bes F. Nowozin C. Appelhoff S. Wahnschaffe A. (2017). Can short-wavelength depleted bright light during single simulated night shifts prevent circadian phase shifts? *Appl. Ergon.* 61 22–30. 10.1016/j.apergo.2016.12.014 28237017

[B51] Ruby N. F. Brennan T. J. Xie X. Cao V. Franken P. Heller H. C. (2002). Role of melanopsin in circadian responses to light. *Science* 298 2211–2213. 10.1126/science.1076701 12481140

[B52] Ruger M. St Hilaire M. A. Brainard G. C. Khalsa S. B. Kronauer R. E. Czeisler C. A. (2013). Human phase response curve to a single 6.5 h pulse of short-wavelength light. *J. Physiol.* 591 353–363. 10.1113/jphysiol.2012.239046 23090946 PMC3630790

[B53] Ruifrok A. C. Johnston D. A. (2001). Quantification of histochemical staining by color deconvolution. *Anal. Quant. Cytol. Histol.* 23 291–299. 11531144

[B54] Rupp A. C. Ren M. Altimus C. M. Fernandez D. C. Richardson M. Turek F. (2019). Distinct ipRGC subpopulations mediate light’s acute and circadian effects on body temperature and sleep. *eLife* 8:e44358. 10.7554/eLife.44358 31333190 PMC6650245

[B55] Santhi N. Duffy J. F. Horowitz T. S. Czeisler C. A. (2005). Scheduling of sleep/darkness affects the circadian phase of night shift workers. *Neurosci. Lett.* 384 316–320. 10.1016/j.neulet.2005.04.094 15919151

[B56] Santhi N. Horowitz T. S. Duffy J. F. Czeisler C. A. (2007). Acute sleep deprivation and circadian misalignment associated with transition onto the first night of work impairs visual selective attention. *PLoS One* 2:e1233. 10.1371/journal.pone.0001233 18043740 PMC2077929

[B57] Santhi N. Thorne H. C. Van Der Veen D. R. Johnsen S. Mills S. L. Hommes V. (2012). The spectral composition of evening light and individual differences in the suppression of melatonin and delay of sleep in humans. *J. Pineal Res.* 53 47–59. 10.1111/j.1600-079X.2011.00970.x 22017511

[B58] Sasseville A. Hebert M. (2010). Using blue-green light at night and blue-blockers during the day to improves adaptation to night work: a pilot study. *Prog. Neuropsychopharmacol. Biol. Psychiatry* 34 1236–1242. 10.1016/j.pnpbp.2010.06.027 20599459

[B59] Sasseville A. Paquet N. Sevigny J. Hebert M. (2006). Blue blocker glasses impede the capacity of bright light to suppress melatonin production. *J. Pineal Res.* 41 73–78. 10.1111/j.1600-079x.2006.00332.x 16842544

[B60] Skene D. J. Skornyakov E. Chowdhury N. R. Gajula R. P. Middleton B. Satterfield B. C. (2018). Separation of circadian- and behavior-driven metabolite rhythms in humans provides a window on peripheral oscillators and metabolism. *Proc. Natl. Acad. Sci. U.S.A.* 115 7825–7830. 10.1073/pnas.1801183115 29991600 PMC6065025

[B61] Smith M. R. Cullnan E. E. Eastman C. I. (2008). Shaping the light/dark pattern for circadian adaptation to night shift work. *Physiol. Behav.* 95 449–456. 10.1016/j.physbeh.2008.07.012 18675836

[B62] Souman J. L. Borra T. De Goijer I. Schlangen L. J. M. Vlaskamp B. N. S. Lucassen M. P. (2018). Spectral tuning of white light allows for strong reduction in melatonin suppression without changing illumination level or color temperature. *J. Biol. Rhythms* 33 420–431. 10.1177/0748730418784041 29984614

[B63] St Hilaire M. A. Gooley J. J. Khalsa S. B. Kronauer R. E. Czeisler C. A. Lockley S. W. (2012). Human phase response curve to a 1 h pulse of bright white light. *J. Physiol.* 590 3035–3045. 10.1113/jphysiol.2012.227892 22547633 PMC3406389

[B64] Takahashi J. S. Decoursey P. J. Bauman L. Menaker M. (1984). Spectral sensitivity of a novel photoreceptive system mediating entrainment of mammalian circadian rhythms. *Nature* 308 186–188. 10.1038/308186a0 6700721

[B65] Thapan K. Arendt J. Skene D. J. (2001). An action spectrum for melatonin suppression: evidence for a novel non-rod, non-cone photoreceptor system in humans. *J. Physiol.* 535 261–267. 10.1111/j.1469-7793.2001.t01-1-00261.x 11507175 PMC2278766

[B66] Tischkau S. A. Mitchell J. W. Tyan S. H. Buchanan G. F. Gillette M. U. (2003). Ca2+/cAMP response element-binding protein (CREB)-dependent activation of Per1 is required for light-induced signaling in the suprachiasmatic nucleus circadian clock. *J. Biol. Chem.* 278 718–723. 10.1074/jbc.m209241200 12409294

[B67] Tsai J. W. Hannibal J. Hagiwara G. Colas D. Ruppert E. Ruby N. F. (2009). Melanopsin as a sleep modulator: circadian gating of the direct effects of light on sleep and altered sleep homeostasis in Opn4(-/-) mice. *PLoS Biol.* 7:e1000125. 10.1371/journal.pbio.1000125 19513122 PMC2688840

[B68] van der Lely S. Frey S. Garbazza C. Wirz-Justice A. Jenni O. G. Steiner R. (2015). Blue blocker glasses as a countermeasure for alerting effects of evening light-emitting diode screen exposure in male teenagers. *J. Adolesc. Health* 56 113–119. 10.1016/j.jadohealth.2014.08.002 25287985

[B69] von Gall C. Noton E. Lee C. Weaver D. R. (2003). Light does not degrade the constitutively expressed BMAL1 protein in the mouse suprachiasmatic nucleus. *Eur. J. Neurosci.* 18 125–133. 10.1046/j.1460-9568.2003.02735.x 12859345

[B70] Wakamatsu H. Takahashi S. Moriya T. Inouye S. T. Okamura H. Akiyama M. (2001). Additive effect of mPer1 and mPer2 antisense oligonucleotides on light-induced phase shift. *Neuroreport* 12 127–131. 10.1097/00001756-200101220-00033 11201072

[B71] Warman V. L. Dijk D. J. Warman G. R. Arendt J. Skene D. J. (2003). Phase advancing human circadian rhythms with short wavelength light. *Neurosci. Lett.* 342 37–40. 10.1016/s0304-3940(03)00223-4 12727312

[B72] Yan L. Okamura H. (2002). Gradients in the circadian expression of Per1 and Per2 genes in the rat suprachiasmatic nucleus. *Eur. J. Neurosci.* 15 1153–1162. 10.1046/j.1460-9568.2002.01955.x 11982626

[B73] Yan L. Silver R. (2004). Resetting the brain clock: time course and localization of mPER1 and mPER2 protein expression in suprachiasmatic nuclei during phase shifts. *Eur. J. Neurosci.* 19 1105–1109. 10.1111/j.1460-9568.2004.03189.x 15009158 PMC3271804

[B74] Yan L. Takekida S. Shigeyoshi Y. Okamura H. (1999). Per1 and Per2 gene expression in the rat suprachiasmatic nucleus: circadian profile and the compartment-specific response to light. *Neuroscience* 94 141–150. 10.1016/s0306-4522(99)00223-7 10613504

[B75] Zeitzer J. M. Kronauer R. E. Czeisler C. A. (1997). Photopic transduction implicated in human circadian entrainment. *Neurosci. Lett.* 232 135–138. 10.1016/s0304-3940(97)00599-5 9310298

[B76] Zeitzer J. M. Ruby N. F. Fisicaro R. A. Heller H. C. (2011). Response of the human circadian system to millisecond flashes of light. *PLoS One* 6:e22078. 10.1371/journal.pone.0022078 21760955 PMC3132781

